# Richmond Academy of Medicine and Surgery

**Published:** 1895-03

**Authors:** Mark W. Peyser

**Affiliations:** Secretary; Richmond, Va.


### RICHMOND ACADEMY OF MEDICINE AND SURGERY.

RICHMOND, VA., January 8, 1895.

*Dr. Wm. J. Gordon, President, in the Chair.*

DR. HUGH M. TAYLOR read a paper on the "Surgical Treatment of Spinal Traumatisms." It is necessary for us first to classify what we are going to treat, and then determine how to treat it. The classification I shall make is: 1st. Anatomical, as traumatisms of the cord, membranes, bony canal, and ligaments. 2d. Clinical or pathological; concussion (sic) of the spine, contusions, hemorrhages, inflammatory deposits, cicatricial contractions and morbid changes incident to extra and intra-dural injuries, fractures, dislocations, and the train of symptoms following injuries to the nerves after they have left the cord.

In order to apply intelligent treatment, it is necessary to differentiate between the manifestations of the different varieties of traumatisms. It must be kept clear that all the manifestations to treat are results of pressure, as from effusions of lymph, serum, or blood; clot, bony fragments, cicatricial contraction, etc.

According to most authorities, concussion of the spine is a misnomer, as the cord is so well protected by a water-bath, by being anchored by the nerves in the bony cavity which is situated deep, lying in a mass of muscular tissue and held strongly together by ligaments. Injury while upright is rare, it occurring when the spine is flexed, as in the bending posture. It is hard to realize that with these natural protections there can be such a condition as concussion. It is really a part of shock. If the symptoms extend beyond the time in which shock should be recovered from, contusion is present.

Take into consideration the important part that the ligaments play in traumatisms of the spine; compare sprain of the spine with those of other parts, and we must conclude that sprains of the spine are frequent. Many of the so-called injuries to the spine are ligamentous and not nervous. If a spinal ligament is torn or contused, it must be repaired by new tissue formation, which may affect probably the nerves passing through, causing eneuritis, and trophic, motor, or sensory disturbances. The damage may be outside and yet be manifested as above—*e. g.*, long-continued neuralgia. Many of the so-called railway sprains are simply such.

We do not often have the opportunity of studying contusions of the cord by post mortems. We must study them by analogy, knowing all the results due to pressure.

The most common morbid condition is hemorrhage, the cord being richly supplied with blood-vessels; and a contusion causing their rupture is not by any means rare.

It is important to differentiate pressure due to blood, serum, or lymph. If the symptoms come on immediately, they are the result of fracture or dislocation; if in two or three hours, they are the result of pressure from hemorrhage; if in a day or two, serum is the cause; and if the symptoms of compression occur after weeks, they are due to lymph effusion.

The manifestations of pressure are: 1st. Of the cord proper, depending on the part upon which pressure is exerted. 2d. Of the spinal nerves, as interference with or destruction of their functions, as spasmodic contraction of the muscles supplied, paresis, paralysis, anesthesia, hyperesthesia, formication, etc. The symptoms are not only local, but remote; not only immediate, but subsequent.

It is not easy to say that the damage to a nerve is within or without the cavity. The reflex effects of nerve irritation are manifested at times when no plain explanation can be given. Mitchell and others say they are due to paresis of the higher centers.

*Treatment:* The field of surgical treatment is limited, as when pressure from exudate exists. Traumatisms of the ligaments are not within surgical limits. Massage and extension should be employed for these. For damage to the cord, iodides, mercury, rest, douche, massage, and moral treatment, which is important.

The only classes of injuries calling for surgical treatment are fractures and dislocations. Cut down on the spine to remove fragments and relieve pressure; but this is not an easy matter. It is advised to bite off the spinous and then the transverse process until the cord is reached, and then be guided by indications as to the pressure of clot or effusion by the bulging, discoloration, etc. It is conceded that the danger from hemorrhage or loss of spinal fluid is not as great as that of pressure.

Another method of treatment is absolute rest, extension to head and feet, and the use of sand-bags.

The bladder must be cared for and bed-sores prevented. The reduction of fractures before operation is debatable, on account of the injury that may be inflicted in the process; and then, it is doubtful if fracture or dislocation can be diagnosed without cutting down. MARK W. PEYSER, M.D., Secretary.

---

### SUGAR IN URINE.

Observation has led me to the conviction that, for the general practitioner, the best every-day test for sugar is what is known as Moore's test, namely, boiling the urine with about one-half its volume of a strong solution of caustic potash or soda. By this means relatively small quantities of glucose may be detected; for if the solution is as strong as one part of caustic soda to eight parts of water, it will disclose the presence of 0.1 per cent. by the production of a distinct canary-yellow color; while 1 per cent. of sugar will be shown by the production (on waiting a minute or so) of an almost or quite black color. This is a very simple and economical way of testing the urine for sugar, and one that is entirely sufficient for clinical needs.—DR. C. W. DULLES, in *Philadelphia Polyclinic.*

---

### GELATIN CAPSULES.

There are few conditions of the economy, either in health or disease, when Gelatin Capsules are not available as a means of elegant and palatable medication. Even when the stomach is lacking in secretion a swallow of water will insure its speedy disintegration.

